# SP1-Induced Upregulation of lncRNA LINC00659 Promotes Tumour Progression in Gastric Cancer by Regulating miR-370/AQP3 Axis

**DOI:** 10.3389/fendo.2022.936037

**Published:** 2022-07-26

**Authors:** Yao Wang, Yuanyuan Guo, Tianchi Zhuang, Ting Xu, Minghui Ji

**Affiliations:** ^1^ Department of General Surgery, First Affiliated Hospital, Nanjing Medical University, Nanjing, China; ^2^ School of Medicine Holistic Integrative Medicine, Nanjing University of Chinese Medicine, Nanjing, China; ^3^ School of Nursing, Nanjing Medical University, Nanjing, China

**Keywords:** LncRNA LINC00659, miR-370, AQP3, gastric cancer, metastasis, prognosis

## Abstract

Growing evidence demonstrates that long noncoding RNAs (lncRNAs) play critical roles in various human tumors. LncRNA LINC00659 (LINC00659) is a newly identified lncRNA and its roles in tumors remain largely unclear. In this study, we elucidated the potential functions and molecular mechanisms of LINC00659 on the biological behaviors of gastric cancer (GC), and also explored its clinical significance. We firstly demonstrated that LINC00659 levels were distinctly up-regulated in both GC specimens and cells using bioinformatics analysis and RT-PCR. The results of ChIP assays and luciferase reporter assays confirmed that upregulation of LINC00659 was activated by SP1 in GC. Clinical assays revealed that higher levels of LINC00659 were associated with TNM stage, lymphatic metastasis, and poorer prognosis. Moreover, LINC00659 was confirmed to be an independent prognostic marker for the patients with GC using multivariate assays. Lost-of-function assays indicated that knockdown of LINC00659 suppressed the proliferation, metastasis, and EMT progress of GC cells *in vitro*. Mechanistic investigation indicated that LINC00659 served as a competing endogenous RNA (ceRNA) for miR-370, thereby resulting in the upregulation of leading to the depression of its endogenous target gene AQP3. Overall, our present study revealed that the LINC00659/miR-370/AQP3 axis contributes to GC progression, which may provide clues for the exploration of cancer biomarkers and therapeutic targets for GC.

**Graphical Abstract:**
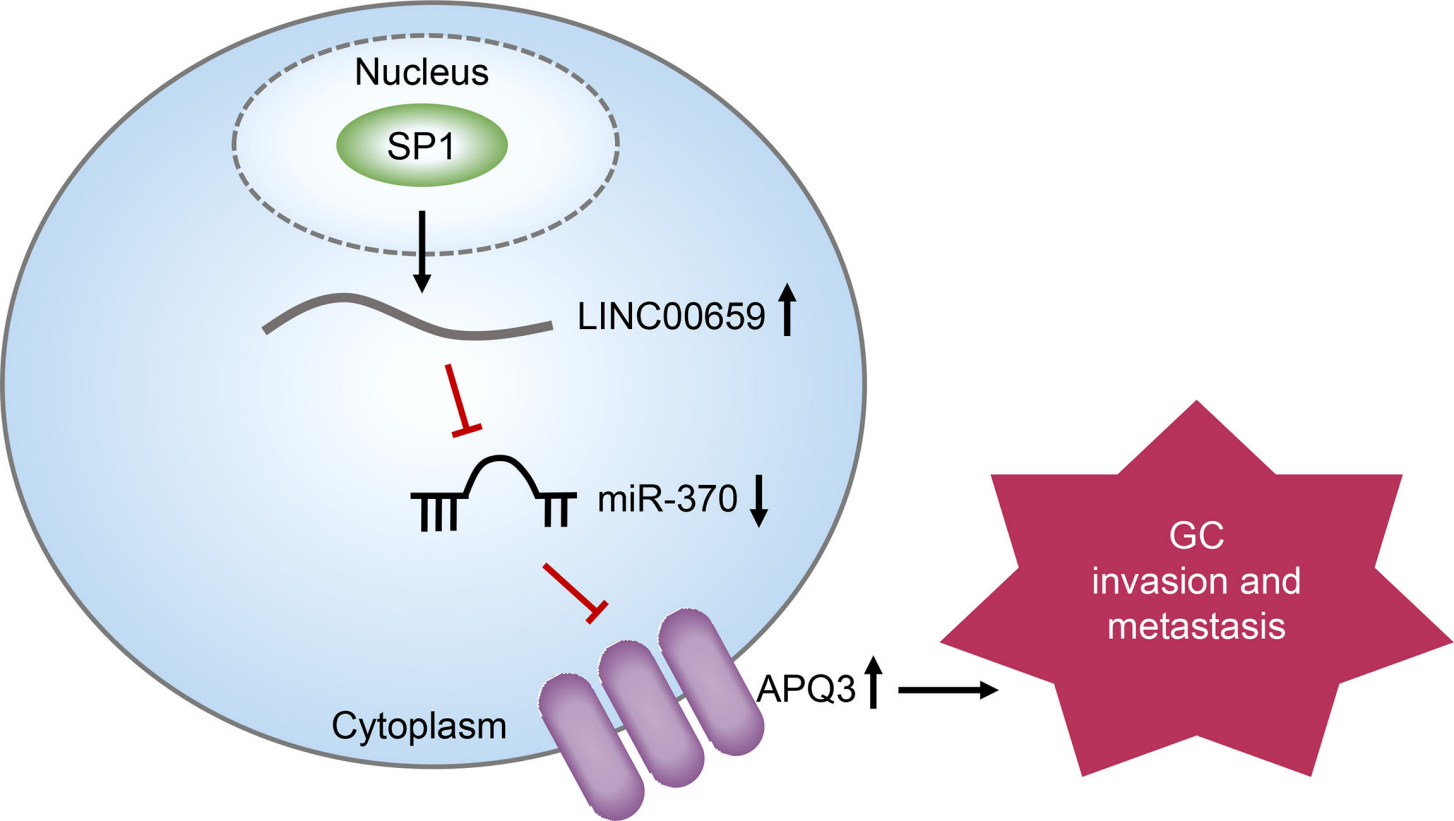


## Introduction

Gastric cancer (GC) is one of the most common gastrointestinal neoplasms and is the 3rd leading cause of tumor-associated mortality worldwide (1). Over the last decades, the incidence of GC is rising steadily in China due to the habits and customs and other unclear risk factors (2). Although therapeutic advancements have been achieved in recent years, the long-term overall survival of GC patients remains poor, and even resectable disease has a 45-85% risk of recurrence (3, 4). The majority of GC deaths are caused by tumor metastasis and patients with advanced stages exhibit a greater chance of developing distant metastasis (5, 6). Up to date, the molecular mechanisms involved in the tumor progression remain largely unclear. Thus, the identification of novel molecules is an urgent need for the advancement of diagnostic progress and the powerful therapy for GC.

Recent evidences indicate that > 85% of the genomes are transcribed as non-coding RNAs (7). Long noncoding RNAs (lncRNAs) are a class of noncoding RNAs > 200 nucleotides in length, with limited protein-coding potentials due to the deficiency of an open reading frame (8). Growing studies proved that lncRNAs play imperative roles in elementary cellular functions, such as cell growth, differentiation, protecting against tissue damage, and tumor progress (9, 10). Importantly, lncRNAs were reported to serve as either oncogene or tumor suppressor genes to participate in the regulation of many kinds of diseases including cancers (11, 12). Recently, a novel hypothesis describes that some functional lncRNAs act as ceRNA by shared miRNA response elements, which expands the field involved in the function of lncRNA in biological progress (13). Previously, several functional lncRNAs have been identified in various cancer, including gastric cancer (14, 15). However, the expression and underlying mechanism of gastric cancer associated with aberrant lncRNAs remain largely unclear.

LncRNA LINC00659 (LINC00659), a recently identified lncRNA, was firstly reported to be highly expressed in colorectal cancer (16). Functionally, this lncRNA was found to accelerate cell apoptosis in colon cancer cells *via* modulating PI3K-AKT signaling. However, the expression and function of LINC00659 on other tumors have not been investigated. In this study, our experiments confirmed that LINC00659 levels were frequently upregulated in GC. Then, we explored the clinical implication and functions of LINC00659 in GC. Our data revealed that the LINC00659 may serve as a potential marker and therapeutic target for GC.

## Materials and Methods

### Clinical Samples

One hundred and twenty GC tumor specimens and adjacent normal samples were collected from patients treated in the First Affiliated Hospital of Nanjing Medical University from January 2016 to December 2018. The samples were frozen using liquid nitrogen, followed by being stored at -80°C until use. Before surgery, the patients received no treatment for anti-cancer. The written informed consent from the patients had been obtained before specimen collection. The procedure was approved by the First Affiliated Hospital of Nanjing Medical University.

### Cell Transfection

GES-1 cells (as control cells) and GC cells (BGC-823, SNU-601, MGC-803 and SGC-7901 cells) were bought from BeiNa Biological corporation (Suzhou, Jiangsu, China). The cells were grown using RPMI-1640 media (Longshang, Technology, Haidian, Biejing, China) containing 10% FBS at 37°C in a 5% CO_2_ incubator. The siRNAs against LINC00659 (lnc-siRNA-1, lnc-siRNA-2), miR-370 mimics or inhibitors were bought from Jima Biological company (Suzhou, Jiangsu, China). LINC00659 and aquaporin 3 (AQP3) overexpressing plasmids (ov-LINC00659, ov-AQP3) were constructed by Meilan Biological corporation. Cell transfection was conducted using Lipofectamine 2000 kits (Dongjun, Qingdao, Shandong, China) based on the guide of users.

### Real-Time PCR

RNAs were extracted based on the protocols of TRIzol reagents (CWBio, Suzhou, Jiangsu, China). Afterwards, the cDNA synthesis was performed using cDNA synthesis kits (Takara, Dalian, China). And then the qPCR detection for LINC00659 and AQP3 was carried out using SYBR Green qPCR kits (Haihong, Ningbo, Zhejiang, China). The reaction conditions were as follows: 95°C for 5 min and 39 cycles of 95°C for 20 s, followed by 72°C for 3 min. For miR-370 detection, we used Transgen two-step miRNA qPCR detecting kits (Tengjun, Changsha, Hunan, China) by the kits’ protocols. The miRNA qPCR conditions were as follows: 95°C for 2 min, and 39 cycles of 95°C for 5 s and 60°C for 30 s. The fold changes of LINC00659, AQP3 and miR-370 were calculated by 2^−△△Ct^ methods. For the detection for LINC00659 and AQP3, GAPDH was used as an internal control and U6 was used as an internal reference for miR-370 measurement. The primers used in the present study were shown in **Table 1**.

**Table 1:** Primers used for qPCR in this study.

Names	Sequences (5’-3’)
LINC00659: F	ACCCCTGAAGGACCATATCCA
LINC00659: R	GGCTCGGCTGTGTCTCAAG
SP1: F	AGTTCCAGACCGTTGATGGG
SP1: R	GTTTGCACCTGGTATGATCTGT
miR-370: R	TGTGAGCCTGCTGGGGTGGA
miR-370: F	GGCCAACCGCGAGAAGATGTT
AQP3: R	GGGGAGATGCTCCACATCC
AQP3: F	AAAGGCCAGGTTGATGGTGAG
GAPDH: F	CAATGACCCCTTCATTGACC
GAPDH: R	ACAAGCTTCCCGTTCTCAG
U6: F	CTCGCTTCGGCAGCACA
U6: R	AACGCTTCACGAATTTGCGT

### Western Blot

Cell lysates from treated GC cells were prepared using RIPA buffer (Dongjun, Qingdao, Shandong, China). Then, protein concentrations were examined by BCA kits (Kedun, Wuhan, Hubei, China). Afterwards, Equal quantities of proteins were mixed with 2 × loading buffer, followed by being separated using 10% SDS-PAGE. The proteins were then transferred onto PVDF membranes. The membranes were then blocked using 5% BSA solution and subsequently incubated with the primary antibodies: anti-E-cadherin antibody (1: 800; CST, Danvers, MA, USA); anti-vimentin antibody (1: 700; PTG, Wuhan, Hubei, China), for 12 h at 4°C. On the second day, after the membranes were washed using TBST buffer, they were probed with corresponding secondary antibodies. Finally, the proteins were measured by ECL kits (Beyotime, Haimen, Jiangsu, China).

### CCK-8 Assay

CCK-8 assays were performed to determine the cell growth using Dojindo cell counting kit-8 kits (Junlong, Wuhan, Hubei, China). Briefly, GC cells after LINC00659 siRNAs transfection were collected and re-placed into plates (96-well; 2 × 10^3^ cells/well). After the cells were attached, 15 microliters of CCK-8 solution were placed into each well, followed by being incubated for 2.5 h. Cell proliferation was determined across 4 days (0, 1, 2, 3 and 4 days). At each time point, the absorbance at 450 nm was examined by a microplate reader.

### EdU Assay

The cellular proliferation was evaluated by EdU assays using Cell-Light EdU kits (Ruibo, Guangzhou, Guangdong, China). In brief, GC cells after LINC00659 siRNAs transfection were placed in plates (48-well) and cultured for 48 h. Subsequently, the cells were treated with EdU solution (100 μl, 50 µM) for 2.5 h, followed by being fixed using paraformaldehyde (4%). Afterwards, the cells were sealed with Apollo Dye reagents, followed by being treated using DAPI solution. The fluorescence was photographed by a fluorescence microscope.

### Colony Formation Assay

LINC00659 siRNAs-transfected GC cells were placed into plates (six-well; 800 cells/well). They were maintained in a 5% CO_2_ incubator at 37°C for about 15 days. After that, the clones were visible and PBS was used for washing. Then, the clones were treated using paraformaldehyde (4%; 10 min), followed by being treated using crystal violet (0.3%) for 15 min. The colonies were imaged using a microscope after they were washed twice by PBS buffer.

### Flow Cytometry Analyses

BD Pharmingen PI/Annexin V-FITC apoptosis detection kits (Yujun, Jinan, Shandong, China) were used for GC cells apoptosis detection. In brief, after GC cells were treated with LINC00659 siRNAs for 48 h, they were harvested and re-placed in 300 μl binding buffer. Thereafter, Annexin V-FITC reagents (5 μl) and PI (5 μl) were put into the cells, followed by being incubated in the light-proof condition at 4°C for 20 min. The samples were then washed and analyzed using a flow cytometer.

### Caspase 3/9 Activity Detection

Beyotime caspase 3/9 activity detection kits were applied for evaluating caspase 3/9 activities in GC cells after treatment. In brief, the treated-GC cells were collected and washed using PBS, followed by adding Lysis Buffer into the cells. After incubation at 4°C for 35 min, the supernatants were obtained by centrifuging the cell lysates (12,000 ×g, 12 min, 4°C), followed by being added with Ac-DEVD-pNA buffer (15 µl). OD405 nm absorbance was measured by a microplate reader after incubation for 2.5 h.

### Wound-Healing Assay

GC cells after LINC00659 siRNAs transfection were placed in plates (twelve-well) at high density. After attachment, the cells (near 100% cell confluence) were scratched by 200 μl pipette tips. The dead cells and debris were washed using PBS buffer. The representative images of wound closures were obtained by imaging at 0 h and 48 h after wound injury.

### Transwell Assay

Transwell assays were conducted using BD Biosciences transwell inserts (8-μm; Ruike, Changsha, Hunan, China). The inserts were pre-coated using Matrigel (70 μl; 1 μg/μl). Thereafter, the LINC00659 siRNAs-treated GC cells (1.5 × 10^5^ cells) were digested and collected in 250 μl serum-free media. The cells were then placed into the inserts and 700 μl media containing 15% FBS were added to the lower transwells. After incubation for 24 h at 37°C, the invasive GC cells at the reverse sides of the membranes were treated using paraformaldehyde (4%) and crystal violet (0.3%) for 15 min. The stained cells were then washed using PBS for three times, and the representative images of the GC cells were photographed by a microscope.

### Subcellular Fractionation Assay

The localization of LINC00659 was determined by using the Thermo Fisher Scientific PARIS kits (Boyuan, Chengdu, Sichuan, China). The nuclear and cytoplasm fractions were respectively isolated according to the kits’ protocols. Subsequently, RNAs were isolated from the cytoplasm and nuclei, respectively. The expression of LINC00659 in cytoplasm and nuclei were examined by qPCR analyses as described above. U6 and GAPDH served as the nucleus and cytoplasmic control, respectively.

### ChIP Assay

Millipore EZ ChIP assay kits (Dongfu, Xiamen, Fujian, China) were used for determination of the ChIP assays by the kits’ protocols. Anti‐SP1 primary antibodies (Abcam, Cambridge, MA, USA) were applied for immune-precipitating the chromatins after the DNAs were sheared into 200-500 bp by sonication. IgG was used as a negative control. The enrichments of isolated RNAs were examined by using qPCR analyses as described above.

### RNA-Pull Down

RNA-pull down assays were performed to assess the interaction between LINC00659 and miR-370. The biotin-labeled LINC00659 (LINC00659-biotin) and negative control (NC-biotin) were synthesized by Ruibo Biotechnology corporation (Guangzhou, Guangdong, China). LINC00659-biotin or NC-biotin was then incubated with Invitrogen streptavidin-coupled Dynabeads (Hongda, Ningbo, Zhejiang, China) to generate probe-bound Dynabeads. Afterwards, the GC cells after transfection with LINC00659 siRNAs were harvested and lysed. Then, probe-bound Dynabeads were added into the lysates and the RNA complexes bound to the beads were eluted. Finally, qPCR analyses were used for detecting miR-370 expression.

### Luciferase Reporter Assay

The regions including predicted miR-370 binding site in LINC00659 were inserted into pGL3 vectors and the plasmids were named LINC00659 WT (wild-type), and its corresponding mutant-type (MUT) vectors were named LINC00659 MUT. In addition, the wild-type or mutated-type predicted miR-370 binding site in 3’UTR of AQP3 mRNA was also cloned into pGL3 vectors, and their corresponding luciferase reporter plasmids were named AQP3 WT and AQP3 MUT. In addition, the predicted SP1 binding site 2 (P2) regions were also cloned into pGL3 vectors and named as P2 WT reporters, and its corresponding mutant-type vectors were named as P2 MUT reporters. These constructions were cloned by Kaigong Biological company (Qingdao, Shandong, China). After the GC cells were prepared, these reporters were respectively transfected with miR-370 mimics or control mimics into the cells using Lipofectamine 2000 reagents as described above. The luciferase intensity was determined using Promega dual-luciferase reporter assay kits (Ansheng, Hefei, Anhui, China).

### Statistical Analyses

Results in this research were analyzed using the SPSS 20.0 software (Chicago, IL, USA). Student’s t−test analysis was used for the comparison between two groups. The association between LINC00659 and clinicopathologic features was tested using the chi-square test. The Kaplan-Meier methods with the log-rank test were applied for determining the overall survival. The influence of each variable on survivals was assessed by the Cox regression assays. A probability value of less than 0.05 was considered as significant.

## Results

### LINC00659 Was Up-Regulated in Both GC Specimens and Cell Lines

**Figure 1:**
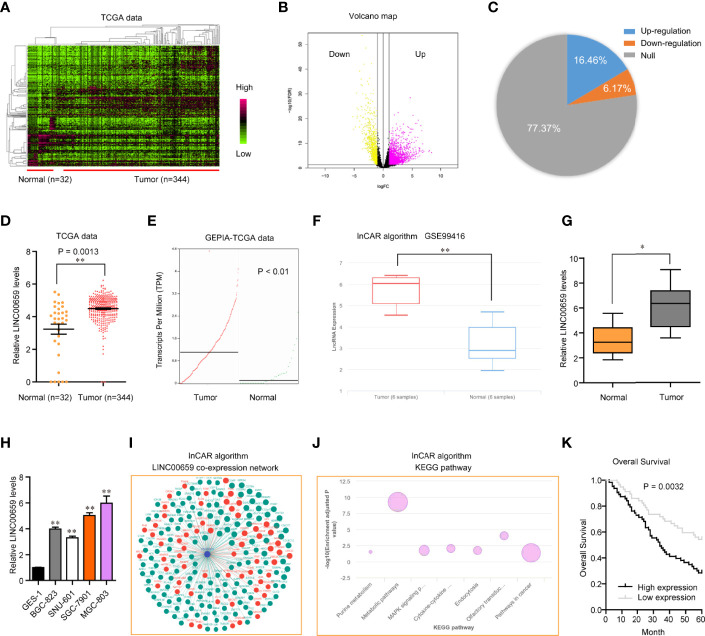
LINC00659 was highly expressed in GC and relevant with poor prognosis. **(A)** Heatmap of TCGA data analysis. **(B)** Volcano map. **(C)** The percentages of up- or down-regulated lncRNAs in GC tumor samples. **(D)** LINC00659 expression in GC tumor and normal samples using TCGA data. **(E)** LINC00659 expression using “GEPIA” algorithm analysis. **(F)** “lnCar” algorithm analyzed LINC00659 expression using GSE99416 data. **(G)** qPCR measured LINC00659 expression in 120 GC tissues. **(H)** qPCR measured LINC00659 levels in GC cell lines. **(I)** “lnCar” algorithm analyzed the co-expression network of LINC00659. **(J)** “lnCar” algorithm analyzed the KEGG pathways of LINC00659. **(K)** Overall survival. *P < 0.05, **P < 0.01.

To discover the potential lncRNAs which might act as key roles in GC tumorigenesis, we conducted bioinformatics analysis using TCGA data. The heatmap and volcano maps of differentially expressed lncRNAs were shown in **Figures 1A, B**. In addition, we found that 16.46% lncRNAs were up-regulated in GC tumor specimens and 6.17% lncRNAs were down-regulated in GC tumor samples (**Figure 1C**). Among these up-regulated lncRNAs, LINC00659, a previously reported onco-promoter, attracted our attention. Indeed, LINC00659 was highly expressed in GC tumor samples (**Figures 1D, E**). Besides, bioinformatics analysis by “lnCar” algorithm using another microarray data (GSE99416) also confirmed that LINC00659 was up-regulated in GC tumor tissues (**Figure 1F**). Data from qPCR also proved that LINC00659 was upregulation in 120 GC tumor specimens (**Figure 1G**). Furthermore, LINC00659 was also highly expressed in GC tumor cells (**Figure 1H**). Next, we employed “lnCar” algorithm to analyze the co-expression network of LINC00659 and found that many genes relevant to LINC00659 were oncogenes (**Figure 1I**). The KEGG pathway analysis using “lnCar” algorithm also indicated that LINC00659 was associated with “pathways in cancer” (**Figure 1J**).

### LINC00659 Up-Regulation Associated With Poor Prognosis of GC Patients

To study the possible roles of LINC00659 on the clinical progress of GC patients, LINC00659 expression levels were classified as low or high based on their median values. The relationships between LINC00659 levels and clinicopathologic features were evaluated. We showed that a high level of LINC00659 was associated with TNM stage (p = 0.013) and lymphatic metastasis (p = 0.017) (**Table 2**). However, no significant difference was observed between LINC00659 expressions and other clinical features. Subsequently, the prognostic values of LINC00659 in GC patients were also investigated using Kaplan-Meier methods. As shown in **Figure 1K**, the data revealed that the overall survivals of patients with high LINC00659 expression were distinctly shorter than those with low LINC00659 expression (p = 0.0032). More importantly, the multivariate Cox regression analyses of overall survivals suggested that LINC00659 expression (HR=2.696, 95% CI: 1.195-4.472, p =0.019) was an independent prognostic indicator for overall survival in patients with GC (**Table 3**).

**Table 2:** Correlation of clinicopathological features of GC with LINC00659 expression levels.

Characteristics	All cases	LINC00659 expression		*p* value
		High	Low	
Age				0.643
≥60	70	38	32	
<60	50	25	25	
Gender				0.949
Male	67	35	32	
Female	53	28	25	
Differentiation				0.308
Well-moderate	70	34	36	
Poor	50	29	21	
Lauren type				0.708
Intestinal	59	32	27	
Diffuse and mixed	61	31	30	
Tumor size				0.112
≥5 cm	71	33	41	
<5 cm	49	30	19	
TNM stage				0.013
I/II	79	35	44	
III/IV	41	28	13	
Lymphatic metastasis				0.017
Negative	82	37	45	
Positive	38	26	12	

**Table 3:** Univariate and multivariate Cox proportional hazard model analysis of overall survival and progression-free survival in GC patients.

Variable	Univariate analysis			Multivariate analysis		
	HR	95% CI	*p*	HR	95% CI	*p*
Age	1.324	0.753-1.892	0.215	–	–	–
Gender	1.446	0.825-2.231	0.189	–	–	–
Differentiation	1.479	0.662-2.371	0.112	–	–	–
Lauren type	0.986	0.572-2.174	0.176	–	–	–
Tumor size	1.357	0.821-2.351	0.117	–	–	–
TNM stage	3.214	1.342-4.889	0.008	2.896	1.218-4.562	0.016
Lymphatic metastasis	3.288	1.572-5.012	0.005	2.996	1.186-4.477	0.013
LINC00659 expression	2.987	1.365-4.782	0.009	2.696	1.195-4.472	0.019

### SP1 Acted as an Activator of LINC00659 Aberrantly High Expression in GC Cells

**Figure 2:**
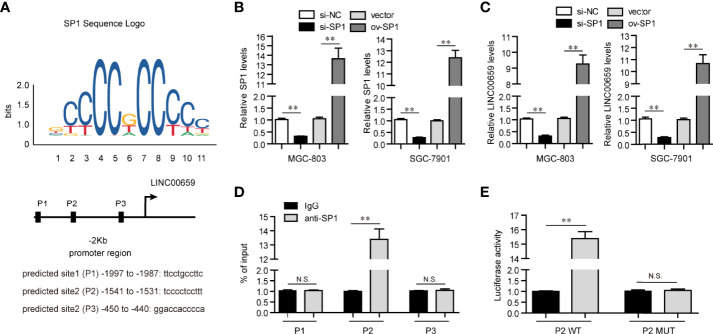
SP1 mediated up-regulation of LINC00659 in GC cells. **(A)** “Jaspar” algorithm predicted SP1 binding sites in LINC00659 promoter sequence. **(B)** qPCR analyses detected SP1 levels in GC cells. **(C)** qPCR analyses detected LINC00659 levels in GC cells. **(D)** ChIP assays. **(E)** Luciferase activity detection. *P < 0.05, **P < 0.01. N.S., no significance.

Mounting evidences had shown that transcription factors (TFs) might play crucial roles in reegulation of lncRNAs expression (17). Therefore, we hypothesized that the specific TF was able to activate LINC00659 aberrantly high expression in GC cells. To achieve that, we first employed “Jaspar” algorithm to predicted the potential TFs which could bind to the promoter region of LINC00659. Among these predicted TFs, SP1, a widely reported TF which induced multiple kinds of lncRNAs aberrant expression, attracted our attention. We selected three predicted potential binding sites across the promoter region of LINC00659 with high predicting scores for further analyzing (**Figure 2A**). The siRNAs against SP1 (si-SP1) and SP1 overexpressing plasmids (ov-SP1) were then also successfully obtained (**Figure 2B**). Next, qPCR analyses revealed that SP1 knockdown or overexpression remarkably decreased or elevated LINC00659 expression, respectively (**Figure 2C**). In addition, we conducted ChIP assays to determine whether SP1 was able to directly target LINC00659 promoter. The results proved that SP1 could directly bind to P2 site of LINC00659 promoter (**Figure 2D**). Besides, luciferase reporter assays further demonstrated that SP1 was capable to target P2 site of LINC00659 promoter (**Figure 2E**). Collectively, our data proved that SP1 activated LINC00659 aberrantly high expression in GC cells *via* binding to its promoter.

### LINC00659 Depletion Suppressed Cellular Proliferation of GC Cells

**Figure 3:**
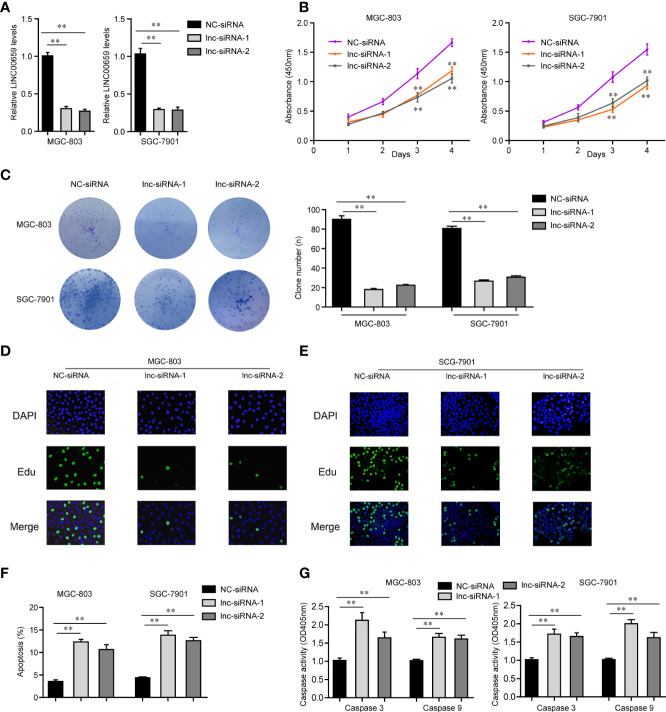
Influence of LINC00659 on GC cell growth and apoptosis. **(A)** qPCR analyses detected LINC00659 levels. **(B)** CCK-8 assays. **(C)** Clonogenic assays. (D, E) EdU assays detected GC cell proliferation. Red represented the proliferation cells. Blue represented the nuclei. **(F)** Flow cytometry analyses examined the apoptosis. **(G)** Caspase 3/9 activity detection. **P < 0.01.

In light of the aberrantly high expressions of LINC00659 in GC specimens and cells, we next attempted to study the possible functions of LINC00659 in GC cells. We first synthesized siRNAs targeting LINC00659 (lnc-siRNA-1, lnc-siRNA-2) and subsequently transfected them into MGC-803 and SGC-7901 cells. After transfection for 48 h, the knockdown efficiency of LINC00659 siRNAs was evaluated by qPCR analyses (**Figure 3A**). Thereafter, CCK-8 assays were carried out to assess the influence of LINC00659 on cellular proliferation. The results demonstrated that knockdown of LINC00659 notably repressed the cell viability of GC cells (**Figure 3B**). Similarly, w using colony formation assays, we also observed that LINC00659 depression dramatically reduced the colony formation capabilities of GC cells (**Figure 3C**). In addition, our group also conducted EdU assays to further determine the effects of LINC00659 on GC cell proliferation. The data proved that LINC00659 depletion remarkably decreased the number of proliferative GC cells (**Figures 3D, E**). Moreover, the impaction of LINC00659 on cell apoptosis was also evaluated. The results of flow cytometry analyses revealed that repressing LINC00659 expression markedly promoted GC cell apoptosis (**Figure 3F**). Besides, mechanical studies showed that LINC00659 deficiency significantly increased caspase 3/9 activities in GC cells (**Figure 3G**). Overall, the data validated that LINC00659 knockdown inhibited GC cell proliferation and promoted cell apoptosis.

### LINC00659 Knockdown Inhibited the Mobility of GC Cells

**Figure 4:**
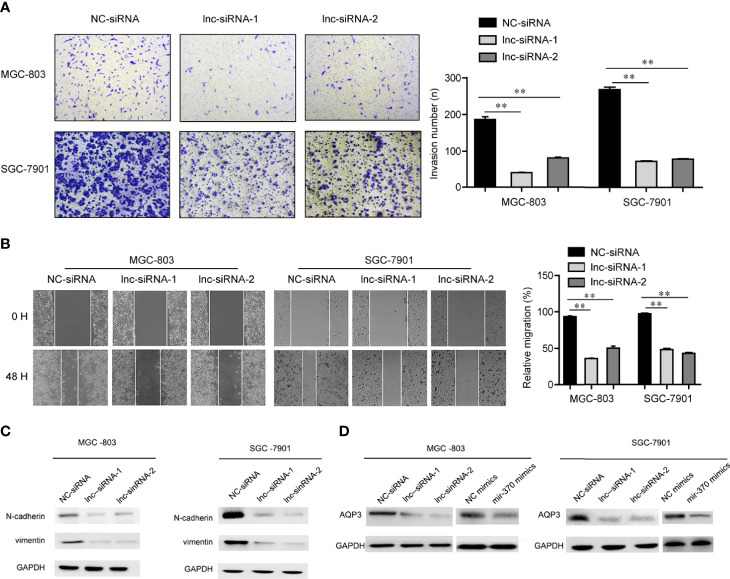
LINC00659 contributed GC cell mobility. **(A)** Transwell assays detected the invasive abilities of GC cells. **(B)** Wound-healing assays determined the cell migration capacities of GC cells. **P < 0.01.

In order to evaluate the functions of LINC00659 on metastasis of GC, the metastasis abilities of GC cells were determined. First, we performed transwell assays to examine the changes of invasion abilities of GC cells after their LINC00659 was knocked down. As the data presented in **Figure 4A**, the invasive cells in the LINC00659-silenced groups were notably fewer than that of the control group, which indicated that depression of LINC00659 significantly reduced the invasion abilities of GC cells. Thereafter, we conducted wound-healing assays to evaluate the effects of LINC00659 silence on GC cell migration. The results validated that LINC00659 knockdown remarkably impaired the relative migratory rates of GC cells (**Figure 4B**). Besides, the mechanical studies revealed that repression of LINC00659 led to markedly decreased protein levels of N-cadherin and vimentin in GC cells (**Figure 4C**). In conclusion, these results suggested that LINC00659 deficiency impeded the metastatic potentials of GC cells through modulating epithelial-mesenchymal transition.

### Screen and Verified MiR-370 Could Directly Target LINC00659

**Figure 5:**
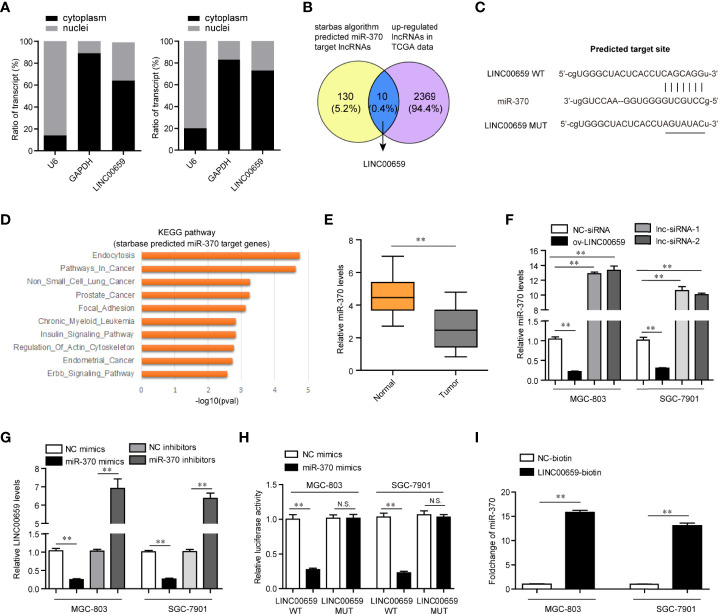
MiR-370 was a target of LINC00659. **(A)** Subcellular fractionation assays. **(B)** The intersection of the predicted miR-370 target lncRNAs and up-regulated lncRNAs in GC tumor specimens. **(C)** “starbase” algorithm predicted the binding site between LINC00659 and miR-370. **(D)** KEGG pathway analysis using the “starbase” algorithm predicted miR-370 target genes. **(E)** qPCR analyses detected miR‐370 expression in 120 GC samples. **(F)** qPCR measured miR‐370 levels in GC cells after various treatment. **(G)** qPCR measured LINC00659 levels in GC cells after various treatment. **(H)** Luciferase activity detection. **(I)** RNA-pull down assays. **P < 0.01. N.S., no significance.

Having proved that depression of LINC00659 suppressed GC tumorigenesis, we next attempted to uncover its detailed molecular mechanisms. Previous reports had validated that lncRNAs (particularly in the cytoplasm) could function as miRNA sponges and subsequently inhibit the functions of the corresponding miRNAs (18). Therefore, we first conducted subcellular fractionation assays to determine the distribution of LINC00659 in GC cells. The data clarified that LINC00659 was mainly located in cytoplasm and thereby it might serve as a miRNA sponge (**Figure 5A**). Hence, we next performed bioinformatics analysis using “starbase” algorithm to obtain the potential target miRNAs of LINC00659. Among these predicted miRNAs, miR-370, a widely reported tumor suppresser in diverse cancer types, attracted our attention. Indeed, the intersection of predicted miR-370 target lncRNAs and up-regulated lncRNAs in GC tumor specimens using TCGA data analysis was also included LINC00659 (**Figure 5B**). Therefore, we next sought to evaluate whether miR-370 was a target of LINC00659 in GC cells. The predicted binding site (wild-type, mutant-type) between miR-370 and LINC00659 was shown in **Figure 5C**. Besides, KEGG pathway analysis using “starbase” algorithm indicated that miR-370 was significantly relevant to “pathway in cancer” and several cancer types (**Figure 5D**). The results from qPCR assays also demonstrated that miR-370 was obvious down-regulation in GC tumor samples (**Figure 5E**). In addition, ectopic expression of LINC00659 was able to reduce miR-370 levels, while LINC00659 deficiency markedly promoted miR-370 expression (**Figure 5F**). Vice versa, the LINC00659 levels were also inhibited or elevated by miR-370 overexpression or knockdown in GC cells (**Figure 5G**). Next, the direct binding between miR-370 and LINC00659 was assessed by luciferase reporter assays. The results validated that co-transfection with LINC00659 wild-type (wt) but not mutant-type (mut) reporters and miR-370 mimics led to remarkably decreased luciferase activities in GC cells (**Figure 5H**). Furthermore, RNA-pull down assays directly proved that LINC00659 could interact with miR-370 in GC cells (**Figure 5I**). Taken together, our data demonstrated that miR-370 could directly target LINC00659.

### AQP3 Was a Target of miR-370 and LINC00659 Regulated miR-370/AQP3 Axis in GC Cells

**Figure 6:**
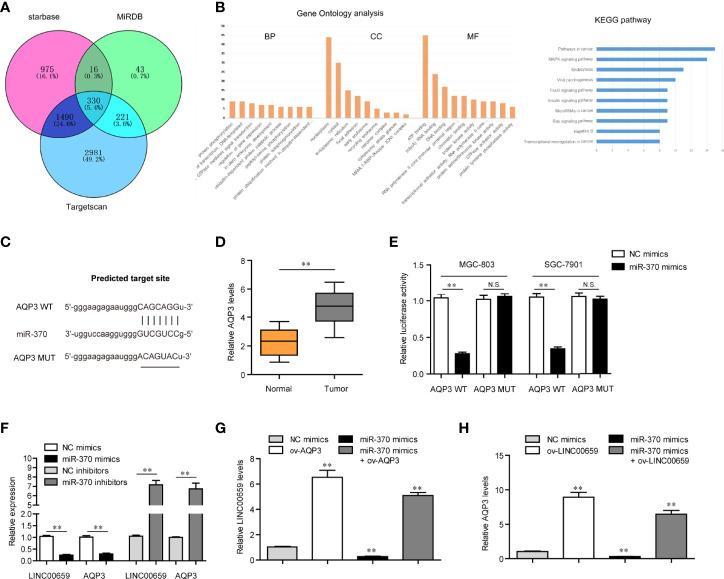
LINC00659 modulated AQP3 expression *via* sponging miR-370 in GC cells. **(A)** Venn diagram of common target genes using three classical predicting algorithms. **(B)** Gene ontology (GO) and KEGG pathway analysis of these commonly predicted genes. **(C)** The potential target site for miR-370 in 3’UTR of AQP3 mRNA. **(D)** qPCR detected AQP3 expression in 120 GC samples. **(E)** Luciferase activity detection. **(F)** qPCR analyses detected LINC00659 and AQP3 levels. **(G)** qPCR examined LINC00659 levels in MGC-803 cells after various treatment. **(H)** qPCR determined AQP3 expression in MGC-803 cells after various treatments. **P < 0.01. N.S., no significance.

We next sought to discover the downstream target gene of miR-370. For that purpose, we performed bioinformatics analysis using “starbase”, “miRDB” and “Targetscan” algorithms to predict the potential target genes of miR-370 and found that there were 330 commonly predicted genes (**Figure 6A**). Gene ontology (GO) and KEGG pathway analysis revealed that these commonly predicted genes were associated with cancers (**Figure 6B**). Among these 330 commonly predicted genes, AQP3, one member of the aquaporins family which acted as onto-promoters in diverse cancer types, attracted our attention. The binding site between miR-370 and 3’UTR of AQP3 mRNA was shown in **Figure 6C**. In fact, qPCR analyses validated that AQP3 was dramatically up-regulated in GC tumor samples (**Figure 6D**). Therefore, we next aimed to investigate whether AQP3 was a direct target of miR-370. To achieve that, we conducted luciferase reporter assays and the results certified that ectopic expression of miR-370 notably decreased the luciferase activities in GC cells transfected with AQP3 wild-type (WT) but not mutant-type (MUT) reporter plasmids, which suggested that miR-370 was able to directly target AQP3 (**Figure 6E**). Additionally, miR-370 mimics transfection could markedly reduce both levels of LINC00659 and AQP3, while miR-370 knockdown remarkably elevated LINC00659 and AQP3 expression (**Figure 6F**). Moreover, enhancing AQP3 expression significantly increased LINC00659, and AQP3 overexpression notably abrogated the inhibitory effects of miR-370 on LINC00659 levels (**Figure 6G**). Besides, forced expression of LINC00659 could also elevate AQP3 levels, and enhancing LINC00659 expression was also able to reverse the suppressive influence of miR-370 on AQP3 expression (**Figure 6H**). To sum up, the data suggested that LINC00659 modulated the miR-370/AQP3 axis in GC cells.

## Discussion

The great development of targeted therapies in clinical management has improved the clinical outcome of GC patients. However, sensitive biomarkers which can guide the clinical application of specially targeted therapies are urgently needed (19, 20). Functional lncRNAs may be applied for this problem. In this study, we firstly detected the expression levels of LINC00659 in GC, finding that LINC00659 was distinctly upregulated in GC tissues and cell lines. By analyzing clinical data, the levels of LINC00659 were observed to be distinctly associated with lymphatic metastasis and TNM stage, indicating that LINC00659 may influence the prognosis of GC patients. Further Kaplan-Meier methods revealed that patients with high-LINC00659 levels had shorter overall survival time. More importantly, our group confirmed LINC00659 as an independent prognostic factor for GC patients using multivariate analyses. To our best knowledge, this is the first time to provide the evidence that LINC00659 expression was distinctly upregulated and associated with poor prognosis in GC.

Increasing research indicated that some epigenetic modulators and key transcription factors (TFs) contributed to the dysregulation of lncRNAs in various tumors, such as SP1, STAT3 and E2F1 (21–23). SP1 was identified as a common transcription factor that is dysregulated in many tumor types and contributes to tumor proliferation and metastasis (24). However, its direct target lncRNAs that were involved in tumor progression are not well defined. In this study, we found that LINC00659 expression could be activated by SP1 *via* binding to its promoter region. Our findings suggested that the dysregulated pattern of lncRNAs expression in GC was associated with the modulation of different modulators.

Recently, LINC00659 was demonstrated to be distinctly upregulated in colorectal cancer, which was in line with our findings (16). Functionally, it was observed that knockdown of LINC00659 suppressed colon cancer cell growth and accelerate cell apoptosis *via* modulating PI3K-AKT signaling, suggesting that LINC00659 served as a tumor promoter in colon cancer. Herein, we also performed functional assays to explore the possible roles of LINC00659 in GC cells, finding that silencing LINC00659 distinctly inhibited the proliferation, migration, invasion and EMT progress in GC cells. In addition, the apoptosis ability of GC cells was also accelerated after the suppression of LINC00659. Thus, our findings indicated that higher levels of LINC00659 displayed tumor-promotive roles in GC progress, which was consistent with its function in colon cancer.

Growing studies have recently revealed a different regulatory mechanism between miRNAs and lncRNAs which act as molecular sponges to compete for miRNAs, thus competitively suppressing miRNAs levels (25, 26). For instance, lncRNA LUCAT1 accelerated ovarian cancer progression *via* the modulation of miRNA-612/HOXA13 pathway (27). LncRNA HOTAIR promoted the proliferation and metastasis of GC cells *via* increasing HER2 expressions *via* sponging miRNA-331-3p (28). In this study, the results of RT-PCR assays revealed that LINC00659 was mainly detected in the cytoplasm of GC cells, which highlighted the potential of LINC00659 as a ceRNA. Subsequently, bioinformatics analysis was conducted for the preliminary exploration of miRNAs with complementary base pairing with LINC00659. Among 38 potential miRNAs, miR-370 attracted our attention due to its distinct change in expression after the overexpression of LINC00659. In addition, a luciferase activity assay demonstrated the direct binding relationships between LINC00659 and miR-370. Previously, the downregulation of miR-370 and its anti-oncogenic roles in several tumors have been demonstrated. Ning et al. suggested that miR-370 suppressed the metastasis ability of GC cells *via* regulating PAQR4 (29). Herein, we found that miR-370 had several potential targets which were involved in several tumor-related pathways, highlighting miR-370 as an important regulator in tumor progression. In addition, the interaction between LINC00659 expressions and miR-370 expressions were also confirmed. Thus, these findings indicated that LINC00659 contributed to tumor progression by sponging miR-370.

AQP3, located on 9p13.3, is a member of the aquaporin family of essential transmembrane factors, and its frequent expressions are found in basolateral plasma membranes of multiple human epithelia (30). In recent years, more and more evidence indicated that AQP3 was abnormally expressed in several tumors (31). In addition, the tumor-related functions of AQP3 in tumors have also been reported. Satooka et al. showed that AQP3 overexpression promoted the migration of breast cancer cell by regulating hydrogen peroxide transport (32). Huang et al. showed that AQP3 can promote tumor growth of pancreatic cancer cells *via* regulating mTOR signaling (33). In GC, overexpression of AQP3 and its tumor-promotive roles were also confirmed both *in vitro* and *in vivo* (34). Our previous studies found that AQP3 promotes the invasion and metastasis of gastric cancer cells by regulating epithelial mesenchymal transformation (35). In this study, to further determine the mechanisms involved in the function of miR-370 in GC progress, we searched three databases, finding that AQP3 may be a target of miR-370. The results of Bioinformatics revealed that AQP3 was positively associated with several cancer-related pathways. Luciferase reporter assays demonstrated that miR-370 can reduce the luciferase activity of MGC-803 and SGC-7901 with abundant AQP3-3’ UTR-wt plasmids, suggesting that miR-370 directly targeted AQP3. Moreover, the results of rescue experiments revealed that overexpression of LINC00659 could reduce the mRNA levels of AQP3 which was suppressed by miR-370 mimics. Overall, our findings firstly indicated that LINC00659 served as a tumor promoter in GC by acting as a molecular sponge of miR-370 to modulate AQP3 expression.

## Conclusions

We firstly showedthat SP1-induced upregulation of LINC00659 promoted GC progression by modulating miR-370/AQP3 axis (**Graphical Abstract**). The high levels of LINC006591 were associated with poor clinical outcome in GC patients. Our findings may help to provide new clue for a better understanding of the pathogenesis of GC and explore the feasibility of lncRNAs-directed diagnosis and treatment for GC.

## Data Availability Statement

The original contributions presented in the study are included in the article/supplementary material. Further inquiries can be directed to the corresponding author.

## Author Contributions

YW, YG and TZ contributed to the study conception and design. TX performed the material preparation, data collection, and analysis. YW and MJ wrote the first draft of the manuscript. All authors have read and approved the final manuscript.

## Funding

This work was funded by High School Philosophy and Social Science Research Fund Project, 2019, Jiangsu Province (2020SJA0298), the Natural Science Foundation of Jiangsu province (grant no. BK20180678) the Priority Discipline Development Program of Jiangsu Higher Education Institutions [General Office, the People’s Government of Jiangsu Province(2018)No.87]

## Conflict of Interest

The authors declare that the research was conducted in the absence of any commercial or financial relationships that could be construed as a potential conflict of interest.

## Publisher’s Note

All claims expressed in this article are solely those of the authors and do not necessarily represent those of their affiliated organizations, or those of the publisher, the editors and the reviewers. Any product that may be evaluated in this article, or claim that may be made by its manufacturer, is not guaranteed or endorsed by the publisher.
